# Alternative Splicing Programs in Prostate Cancer

**DOI:** 10.1155/2013/458727

**Published:** 2013-08-01

**Authors:** Claudio Sette

**Affiliations:** ^1^Department of Biomedicine and Prevention, University of Rome “Tor Vergata,” 00133 Rome, Italy; ^2^Laboratory of Neuroembryology, Fondazione Santa Lucia IRCCS, 00143 Rome, Italy

## Abstract

Prostate cancer (PCa) remains one of the most frequent causes of death for cancer in the male population. Although the initial antiandrogenic therapies are efficacious, PCa often evolves into a hormone-resistant, incurable disease. The genetic and phenotypic heterogeneity of this type of cancer renders its diagnosis and cure particularly challenging. Mounting evidence indicates that alternative splicing, the process that allows production of multiple mRNA variants from each gene, contributes to the heterogeneity of the disease. Key genes for the biology of normal and neoplastic prostate cells, such as those encoding for the androgen receptor and cyclin D1, are alternatively spliced to yield protein isoforms with different or even opposing functions. This review illustrates some examples of genes whose alternative splicing regulation is relevant to PCa biology and discusses the possibility to exploit alternative splicing regulation as a novel tool for prognosis, diagnosis, and therapeutic approaches to PCa.

## 1. Introduction

Cancer cells are characterized by uncontrolled growth and ability to migrate from the primary lesion and to establish metastases in distant tissues. Standard therapies involve surgical removal of the tumor mass, radiation, and chemotherapy, which exploit the increased growth rate of cancer cells with respect to surrounding cells. More targeted approaches have also been developed in the last decades by directly inhibiting the function of the oncoproteins responsible for the neoplastic transformation. Nevertheless, although many human cancers initially respond to therapies, and in some cases patients are cured, most of them are characterized by disease relapse that often occurs in more aggressive and incurable forms. In this regard, a clear example of aggressive relapsing tumor is represented by prostate carcinoma (PCa), which remains one of the main causes of death for cancer in the male population [1, 2]. Understanding the mechanisms that lead to the acquisition of resistance to therapies in PCa patients might offer new molecular markers for earlier and more accurate diagnoses. Furthermore, identification of the key players involved in the transition to therapy-refractory stages may shed light on new targets for pharmaceutical intervention and open the path for the development of novel and more efficacious therapies. 

PCa cells rely on androgens and on the androgen receptor (AR) for proliferation [1]. Under normal conditions, the AR is localized in the cytoplasm; upon binding to androgens, the receptor dimerizes, translocates to the nucleus, and trans-activates genes containing androgen-responsive elements in their promoter regions. Clinical treatments are currently based on androgen ablation therapies, obtained by chemical castration with drugs that block secretion of the hormone or by directly targeting the AR with androgen antagonists [1, 2]. However, after initial remission, many patients develop a hormone-resistant or castration-resistant form of PCa (CRPCa), for which no cure is available [1, 2]. Notably, in most cases CRPCa cells still require the AR, but they can bypass activation by androgens or be stimulated by the low androgen levels present during the therapy or by the antagonists used for the therapy [3]. Several mechanisms for the development of androgen insensitivity of the AR have been documented [1]. Among these, recent evidence points to alternative splicing (AS) of AR as a key resource utilized by PCa cells to evade the normal route of activation of this pathway [4]. 

AS is emerging as a key step in the regulation of crucial cellular and developmental pathways in higher eukaryotes [5]. With regard to PCa, it has been proposed that the “splicing signature” represents a more accurate parameter to stratify patients than the “transcriptome signature,” which is typically analysed by conventional microarray analyses [6]. Thus, understanding the regulation of splicing in normal and pathological prostate cells may help identify novel markers and targets for future therapeutic approaches to this neoplastic disease. 

## 2. Alternative Splicing and Cancer

The recent advent of high-throughput RNA sequencing has unveiled new layers of regulation of gene expression and highlighted the extreme complexity and versatility of the genome. The majority of human genes encode multiple transcripts through the use of alternative promoters, AS and alternative polyadenylation [5, 7, 8]. AS is a combinatorial mechanism that expands the coding potential of the genome by allowing the production of protein isoforms with different or even antagonistic functions from a single gene [5, 7, 8]. Splicing is orchestrated by a ribonucleoprotein complex called “spliceosome,” which recognizes exon-intron junctions, excises introns, and ligates exons. The lack of stringent consensus sequences at exon-intron junctions in higher eukaryotes allows flexibility in recognition by the spliceosome. Numerous RNA binding proteins (RBPs) interact with components of the spliceosome and reinforce or weaken recognition of exon-intron junctions. The interplay between these splicing factors determines the choice of variable exons by the spliceosome and causes heterogeneity in pre-mRNA processing [5, 7]. As a consequence, changes in the expression levels or in the activity of splicing factors can selectively influence AS of many genes [5, 7]. Although the flexibility of AS regulation has represented an evolutionary advantage for higher eukaryotes, it also represents a risk factor. In particular, mounting evidence illustrates how defective regulation of AS correlates with onset and progression of human cancers [7, 8]. 

Herein, the literature describing the impact of AS in the onset and progression of PCa will be reviewed. High-throughput analyses of specimens from PCa patients have highlighted more than 200 genes whose AS is differentially regulated in the neoplastic tissue [6], indicating that this mechanism can substantially contribute to the heterogeneity of gene expression in cancer cells. Nevertheless, the physiological consequences of the majority of these aberrant AS events are still unknown and will require direct investigation. This review will focus on the regulation of genes and splicing regulators whose relevance for PCa has been firmly documented.

## 3. The Androgen Receptor 

**Figure 1:**
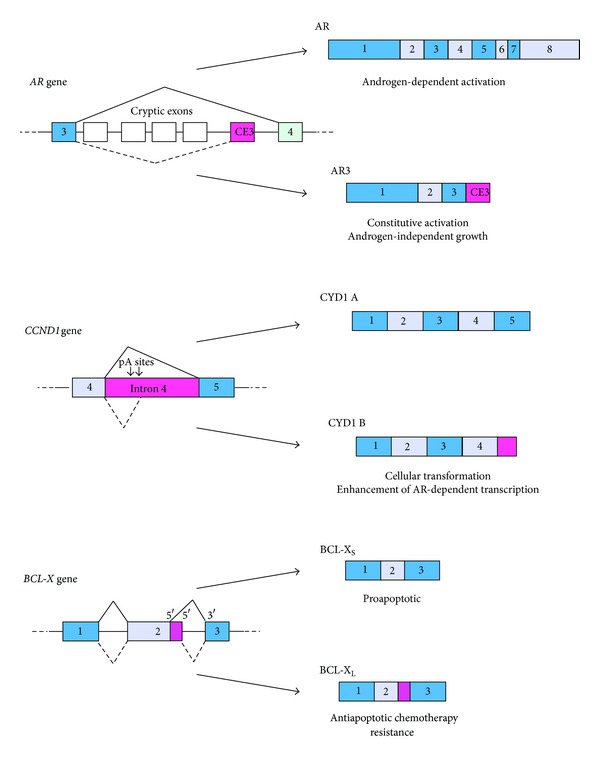
Representative examples of genes whose alternative splicing affects prostate cancer cell biology. The left side of the figure illustrates the genomic structure of the alternatively spliced regions of the *AR*, *CCND1,* and *BCL-X* genes. Solid and dashed lines show the alternative splicing events reported in the literature. On the right side, the alternative variants produced by splicing are shown. The specific features of the protein isoforms produced by alternative splicing are summarized under the scheme of each variant.

Several reports have documented the expression of alternatively spliced AR variants lacking the C-terminal ligand-binding domain of the canonical receptor (reviewed in [4]). Many of these AR splice variants are constitutively nuclear and active even in the absence of androgens (Figure 1), thus indicating their potential role in the acquisition of the CRPCa phenotype [4]. Expression of most of these variants arises from the inclusion of cryptic exons located in intron 2 and 3 of the *AR* gene. The heterogeneity of the AR variants reported in various studies is also due to the frequent amplification of the exon 2-exon 3 genomic region of *AR* [4, 9]. In all reported variants, however, the splicing of these cryptic exons invariably introduces premature stop codons and termination sites, thereby yielding shorter AR proteins of 75–80 kDa, which lack the androgen-binding domain [4]. In some cases, these truncated AR variants can function independently of the full-length AR, and their selective knockdown was shown to block the androgen-independent growth of CRPCa cells while maintaining responsiveness to the hormone [10]. Importantly, expression of an AR variant containing a cryptic exon located in intron 3 (CE3) in clinical PCa specimens positively correlated with poor prognosis after surgery [11]. This variant was also expressed at higher levels in CRPCa with respect to PCa patients [12]. Furthermore, constitutively active truncated AR splice variants were recently shown to confer resistance also to the next generation of AR inhibitors, thus limiting their therapeutic efficacy for many patients [13]. However, since the expression of these shorter AR variants was also observed in normal prostate tissues, it is unlikely that they drive the initial steps of neoplastic transformation, opening the possibility that AS of the* AR* gene plays also a physiological role in the gland [4].

The mechanisms that lead to increased expression of aberrant AR splice variants in PCa are still largely unknown. One possible cause of defective splicing is the alteration of the genomic *AR* locus, which often occurs in CRPCa. For instance, disruption of the *AR* splicing pattern in the 22Rv1 PCa cell line was linked to duplication of the genomic region containing exon 3 and some of the cryptic exons [9]. Alternatively, aberrant expression of specific splicing factors in PCa cells may also contribute to unbalanced splicing and aberrant recognition of cryptic exons in the *AR* gene. Thus, given the strong relevance of these constitutively active *AR* variants for CRPCa progression, further studies elucidating the regulation of their expression are strongly encouraged. 

## 4. KLF6

Kruppel-like factor 6 (KLF6) is a zinc finger transcription factor that is mutated in a subset of human PCas [14, 15]. KLF6 is known to regulate cell proliferation by inducing the expression of the cell cycle inhibitor p21 (WAF1/CIP1). Notably, this effect of KLF6 does not require p53, suggesting that KLF6 is a tumor suppressor gene that functions as a p53-alternative brake for cell cycle progression in normal cells [14]. One of the mutations found in PCa patients consists of a single nucleotide change that creates a binding site for the splicing factor SRSF5 (SRp40) and enhances splicing of three alternative mRNA variants encoding for truncated KLF proteins, named KLF6-SV1, SV2, and SV3 [16]. These splice variants are upregulated in tumor versus normal prostatic tissue. A single G > A mutation in intron 1 was shown to recapitulate the altered splicing pattern of KLF6 when it was introduced in a minigene, and it was found to correlate with worse prognosis in patients [16]. The KLF-SV1 variant was characterized further and shown to function as a dominant-negative protein, which antagonizes the function of full length KLF6, leading to decreased p21 expression and enhanced cell growth [16]. Increased expression of this splice variant in PCa patients predicted poorer outcome after surgery and was associated with development of hormone-refractory metastatic PCa [17]. Furthermore, while knockdown of the full length KLF6 promoted tumor formation in nude mice, selective silencing of the KLF6-SV1 variant inhibited it [18]. Conversely, PCa cells overexpressing KLF6-SV1 are more prone to develop metastases in various organs of the mouse models used in the study [17]. Thus, a mutation affecting *KLF6* AS represents a critical mechanism for the inactivation of a tumor suppressor gene in PCa, suggesting that interfering with this splicing event in PCa cells might restore the growth-inhibitory activity of this transcription factor.

## 5. Cyclin D1

*CCND1* is a protooncogene that encodes for cyclin D1, which associates with the cyclin-dependent kinase 4 (CDK4) to drive progression through the G1 phase of the cell cycle. Importantly, *CCND1* expression is often deregulated in cancer cells [19, 20]. This gene encodes for two alternative transcripts: the common cyclin D1a isoform, containing all five exons, and cyclin D1b, which derives from retention of intron 4 and premature termination of the transcript (Figure 1) [19, 20]. Unlike cyclin D1a, cyclin D1b alone can promote cellular transformation [21], and its expression has been associated with PCa progression and poor prognosis [22]. Interestingly, recent evidence indicated that cyclin D1b promotes AR-dependent transcription of genes involved in PCa metastatic potential, such as the transcription factor SLUG [23]. Cyclin D1a was instead reported to repress the transcriptional activity of AR (Figure 1) [19, 20]. Thus, it is conceivable that a change in the ratio between the cyclin D1 variants will potently enhance hormone-dependent growth of PCa cells.

Given the relevance for PCa cell biology, understanding the regulation of *CCND1* splicing is of crucial importance. It was observed that a polymorphism (G870/A) at the exon 4-intron 4 boundary predisposes cells to cyclin D1b splicing [19, 20]. The splicing factor SRSF1 was shown to bind the exon4/intron4 junction in the nascent *CCND1* pre-mRNA, thereby promoting intron 4 retention and cyclin D1b expression [24]. SRSF1 was hypothesized to favour intron 4 retention by altering exon 4 definition and limiting assembly of the spliceosome at the exon-intron junction [24]. Another splicing factor promoting cyclin D1b expression in PCa cells is SAM68 [25]. In this case, the binding site was identified within intron 4, in proximity of the termination site utilized for the cyclin D1b mRNA. The binding of SAM68 to this region of the pre-mRNA was shown to compete with that of the U1 snRNP [25]. Since deposition of U1 snRNP near cryptic polyadenylation sites located in introns is known to prevent premature termination of transcripts in a genome-wide fashion [26], it is possible that up-regulation of SAM68 unmasks the cyclin D1b termination site by interfering with U1 snRNP binding in intron 4. Notably, both SRSF1 and SAM68 display oncogenic features in several cell types and tissues [7, 27], and their expression positively correlates with that of cyclin D1b in clinical specimens of PCa patients [24, 25]. Thus, it is possible that interfering with the activity of these splicing factors will exert positive effects in therapeutic treatments of PCa through modulation of *CCND1* splicing and expression.

## 6. BCL-X

The *BCL-X* (*BCL2L1*) gene contains 3 exons and encodes for two splice variants [28]. Two alternative 5′ splice sites are present in exon 2 of the gene: selection of the canonical one at the end of the exon yields the long BCL-X_L_ variant, whereas selection of the distal one located 220 bp upstream in the exon produces the short BCL-X_S_ variant. Notably, these two splice variants have opposite effects in the cell, with BCL-X_L_ being prosurvival whereas BCL-X_S_ is proapoptotic (Figure 1) [28]. Thus, regulation of *BCL-X* AS can finely modulate cell viability, illustrating the biological importance of this splicing event. In most cancer cells, including PCa cells, the anti-apoptotic BCL-X_L_ variant is overexpressed and confers resistance to chemotherapeutic treatments [29, 30]. It is predictable that a full understanding of the mechanisms of regulation of *BCL-X* splicing will help develop tools to switch it toward the proapoptotic BCL-X_S_ variant, thereby offering a therapeutic opportunity to sensitize cancer cells to treatments. In line with this notion, treatment of PCa cells with an antisense oligonucleotide (ASO) masking the BCL-X_L_ splice site effectively switched *BCL-X* splicing and induced apoptosis [29]. Interestingly, the proapoptotic effect of the ASO was more pronounced in cancer cells, which display high levels of expression of BCL-X_L_, and it also enhanced their response to chemotherapeutic treatments [30]. Thus, ASOs targeting *BCL-X* splicing may have the advantage of being selective for cancer cells with respect to normal cells, which is a positive feature for an antineoplastic drug. Unfortunately, the delivery of ASOs to cancer cells is still not efficient, thus limiting their application in the clinic, even though development of vehicles favoring their delivery, such as lipid nanoparticles [31], may aid in this direction. 

Although regulation of *BCL-X* splicing is highly relevant to PCa cell biology, not much is known on the mechanism(s) of its regulation in prostate cells. A possible regulator is the protein phosphatase 1 (PP1), whose activity is required for the regulatory effect of ceramide on *BCL-X* splicing [32]. Indeed, PP1 activity was required also for induction of BCL-X_S_ splicing by emetine, a protein synthesis inhibitor, and other proapoptotic drugs in PCa cells [33, 34]. Nevertheless, the mechanism by which PP1 modulates splicing of *BCL-X* is still unknown. PP1 is known to regulate splicing by modulating the activity of splicing factors, either by direct binding to them and regulation of their phosphorylation status [35] or indirectly by regulating kinases involved in their post-translational modifications [36]. Thus, activation of pathways impinging on PP1 may affect *BCL-X* splicing and cell viability through the regulation of the activity of specific splicing factors in PCa cells.

Several splicing factors have been shown to modulate *BCL-X* splicing. Studies performed in a variety of cell models indicated that the heterogeneous nuclear ribonucleoprotein (hnRNP) H and F [37] and the splicing regulators SAM68 [38], RBM25 [39], and RBM11 [40] promote splicing of the proapoptotic BCL-X_S_ variant. By contrast, hnRNP K [41], the serine-arginine (SR) rich proteins SRSF1 [38, 42] and SRSF9 [43], and the splicing factor SAP155 [44] enhance splicing of the anti-apoptotic BCL-X_L_. Which of these factors contribute to the regulation of *BCL-X* splicing in PCa cells is still largely unknown. SRSF1 [24] and SAM68 [45] were shown to be upregulated in PCa and might represent strong candidates for the regulation of this splicing event. Intriguingly, these splicing factors normally modulate *BCL-X* splicing in opposite directions [38]; while the up-regulation of SRSF1 is in line with the high levels of BCL-X_L_ in PCa cells, SAM68 should favour the proapoptotic short variant. However, the splicing activity of SAM68 is finely tuned by phosphorylation [46], and it was shown that tyrosine phosphorylation by the Src-related kinase FYN switched SAM68-dependent splicing of *BCL-X* toward the anti-apoptotic variant [38, 47]. Since tyrosine phosphorylation of SAM68 is increased in specimens of PCa patients [48], it is likely that this RBP can also contribute to the upregulation of BCL-X_L_ in PCa cells. In line with this hypothesis, BCL-X_L_ expression was decreased, and sensitivity to genotoxic agents was increased, after knockdown of SAM68 in the androgen-sensitive LNCaP cell line [45]. 

Thus, based on the observations reported previously, it is predictable that exogenous modulation of *BCL-X* AS through administration of ASOs, or by interfering with the activity of the splicing factors that promote the anti-apoptotic BCL-X_L_ variant, will enhance the efficacy of chemotherapy in advanced PCa, as suggested by preclinical studies in PCa cell lines [30, 31, 45]. 

## 7. TMPRSS2:ERG

ERG is a member of the ETS transcription factor family that is expressed at very low levels in benign prostate epithelial cells. However, PCa patients often carry a fusion of the androgen-responsive *TMPRSS2* gene with *ERG*, which causes aberrantly high expression levels of the transcription factor in the neoplastic cells. A detailed sequencing analysis of the *TMPRSS2:ERG* transcripts isolated from PCa tissues revealed that fusion-derived transcripts underwent profound AS regulation, which yielded mRNA variants encoding both full length ERG proteins and isoforms lacking the ETS domain. Notably, an increase in the abundance of transcripts encoding full length ERG correlated with less favorable outcome in patients [49]. These results support a possible functional role for this transcription factor in PCa pathology and suggest that modulation of AS events promoting less pathogenic variants may produce beneficial effects. 

This hypothesis is also supported by another study that tested the effects exerted by the expression of *TMPRSS2:ERG* alternatively spliced transcripts in an immortalized prostate cell line [50]. It was found that these *TMPRSS2:ERG *splice variants had different oncogenic activities, in terms of promoting proliferation, invasion, and motility. Notably, coexpression of different variants produced stronger effects than either variant alone, suggesting that the presence of several TMPRSS2:ERG isoforms, as it normally occurs in PCa cells, might confer a more malignant phenotype [50]. A further contribution of AS to the heterogeneity of *TMPRSS2:ERG* expression is provided by the extensive variability of the 5′ untranslated region (UTR) in the splice variants observed in patients [51]. Indeed, AS of the 5′ UTR affects the oncogenic potential of the encoded proteins by regulating their translation and activity. Thus, although a functional link between *TMPRSS2:ERG* expression and PCa pathology has not been firmly established yet, this fusion gene appears to be another suitable target for an AS-directed therapeutic approach that would spare normal cells not expressing the chimeric proteins. 

## 8. Splicing Programs in Prostate Cancer

Cancer cells express a number of splice variants that confer them higher resistance to chemotherapeutic drugs and survival advantages. When it was investigated in detail, the specific signature of splice variants expressed by cancer cells has been recognized as a powerful diagnostic and prognostic tool [7, 8]. Even more importantly, the existence of cancer-specific splicing variants of key genes, such as the *AR* or *CCND1* in PCa, might offer a therapeutic opportunity for targeting proteins that are not expressed in healthy cells. For instance, developing tools that specifically modulate the expression of transcript variants preferentially or uniquely produced by cancer cells might slow down tumor growth and/or promote cell death during therapy, while sparing the healthy tissues. Thus, understanding AS regulation at the genome-wide level in PCa cells may not only lead to the identification of novel diagnostic or prognostic biomarkers, but it could also help find tools for novel therapeutic approaches to this neoplastic disease.

A few studies have directly investigated the genome-wide regulation of AS in PCa cell lines and primary tumor tissues. Using a splicing-sensitive microarray, comprising a selected subset of genes and splice variants, it was shown that splicing signatures could efficiently segregate PCa cells lines from cancer cell lines derived from other organs or tissues [6]. Among the alternatively spliced genes, the majority also showed variation in expression levels [6], suggesting that regulation of splicing and transcription were coupled, as also observed in cells exposed to DNA damage [52]. Using the same splicing-sensitive platform, it was also possible to identify splicing signatures that were specific for normal or neoplastic prostate tissues obtained from biopsies [6]. Although this approach was limited to the genes and the splice variants selected for the platform, it provided a first indication that specific changes in splicing occur during prostate tumorigenesis and suggested that splicing variants can represent accurate biomarkers for PCa. Nevertheless, how and when these changes occur, as well as to what extent they contribute to the acquisition of the transformed phenotype, are still open questions. Given the tight association between transcription and splicing, a specific splicing program could result from the different activity of transcription factors, splicing factors, or both. Mounting evidence indicates that all these events contribute to some extent to the acquisition of specific splicing signatures in PCa. 

The most relevant transcription factor involved in PCa is the AR. Several observations suggest that in addition to regulating the expression levels of target genes, AR can also influence the transcript variants encoded by them. Using comprehensive splicing-sensitive arrays, it was demonstrated that stimulation of LNCaP cells with androgens caused qualitative changes in expression of splice variants [53]. Many of the events altered by treatment with androgens were due to usage of alternative promoters within the transcription unit of the target gene. Some of these alternative transcripts were predicted to influence the function of proteins with relevance to PCa, such as the mTOR regulator TSC2. Following androgenic stimulation, AR was recruited to a cryptic promoter upstream of exon 33 in the *TSC2* gene, thereby leading to expression of a truncated transcript lacking the 5′ exons of the gene. This alternative *TSC2* variant would encode a truncated protein lacking the domain required for the interaction with TSC1, which is needed to exert negative regulation of mTOR. Thus, androgens may lead to activation of mTOR by relieving the repressive function of the TSC1/TSC2 complex through AR-dependent induction of a defective variant. It is worthy of notice that activation of the mTOR pathway has been linked to both tumorigenesis and resistance to therapy in PCa [54]. Thus, AR might contribute to prostate tumorigenesis also by causing mTOR activation through expression of this alternative mRNA variant of *TSC2*. 

## 9. Splicing Regulators Contributing to Altered Gene Expression in Prostate Cancer 

**Figure 2:**
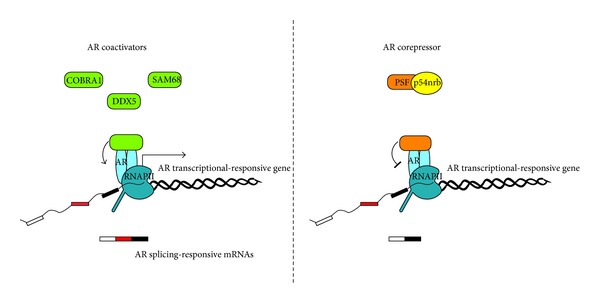
Regulation of cotranscriptional splicing by proteins interacting with the androgen receptor. Coregulators of the androgen receptor (AR) can affect splicing of target genes by direct interaction with AR and modulation of its activity. COBRA1, SAM68, and DDX5 appear to promote the transcriptional activity of AR but differentially act on splicing of variable exons (red box in the left side of the figure); PSF and its interacting protein p54 (right side of the figure) repress the transcriptional activity of AR, but their effect on splicing is currently unknown (see text for more details).

In addition to affecting recruitment of AR to alternative promoters, androgens also affected a number of AS events in several genes [53]. Although the mechanism(s) involved in these events and their potential relevance to PCa biology was not investigated, it might involve the ability of AR to interact with cofactors that modulate the transcriptional elongation rate and/or the recognition of splicing enhancers or silencers in the pre-mRNA (Figure 2). For instance, AR interacts with the cofactor of BRCA1 (COBRA1), and this interaction was shown to influence splicing of the nascent transcripts produced from an androgen-dependent promoter [55]. A similar regulation of AR activity was also documented for the DEAD box RNA helicase p68 (DDX5) in the LNCaP cell line. AR and DDX5 interact and are recruited to the promoter region of the androgen-responsive prostate-specific antigen (PSA) gene. This interaction was functionally relevant, as DDX5 enhanced AR-dependent PSA expression. In addition, by using an AR-dependent minigene reporter, it was shown that DDX5 and AR cooperated in repressing the splicing of variable exons in the *CD44* gene [56]. DDX5 is involved in several steps of co- and posttranscriptional RNA processing, including splicing [57], and some genes appear to be particularly sensitive to the intracellular levels of DDX5 [57, 58]. Hence, since this RNA helicase is upregulated in PCa [56], it will be interesting to determine to what extent it contributes to RNA processing of AR target genes in PCa cells.

AR is also known to interact with several splicing factors, suggesting a direct link between androgen-regulated transcription initiation and pre-mRNA splicing in PCa cells (Figure 2). The PTB-associated splicing factor (PSF) and its cofactor p54nrb participate to androgen-dependent protein complexes containing the AR. PSF and p54nrb inhibit the transcriptional activity of AR by interfering with its binding to androgen response element and by recruiting a histone deacetylase to AR-responsive promoters [59]. Although direct investigation of the effect of these splicing factors on AR-dependent splicing events was not addressed, it is likely that AS is also affected by this interaction. Another splicing factor that may participate to AR-dependent splicing regulation is SAM68 [60], which is frequently upregulated in PCa [25, 45]. SAM68 interacts with AR and is recruited to the PSA promoter [60], like DDX5 [55]. Interestingly, however, the interaction between SAM68 and AR exerted different effects on transcription and splicing, as the two proteins cooperated in transcriptional activation of AR-target genes but opposed each other in splicing of the *CD44 *variable exons from a reporter minigene [60]. Unfortunately, the direct effects of all these RBPs on AR-dependent splicing of endogenous transcripts have not been addressed yet. Nevertheless, it is likely that, depending on the specific complex formed, AR can differentially influence splicing of its target genes in PCa cells. 

An additional layer of regulation of the aberrant splicing program in PCa might rely on the up-regulation of specific splicing factors. Beside the already mentioned SAM68 [25, 45], one likely candidate is SRSF1, a splicing factor that is upregulated in many human cancers and was shown to behave as an oncogene in mice and humans [61]. In cancer cells of other tissues, SRSF1 modulates the expression of splice variants of the *BIN1* and *BIM* genes that lack proapoptotic functions [61, 62]. Moreover, SRSF1 promotes splicing of MNK2b [61], a splice variant of the eIF4E kinase MNK2 that was shown to confer chemoresistance in pancreatic adenocarcinoma cells [63]. Importantly, MNK-dependent phosphorylation of eIF4E strongly contributes to PCa tumorigenesis both *in vitro* and *in vivo* [64, 65], and a tight balance between the MNK/eIF4E and the mTOR pathways is required to maintain efficient protein synthesis in PCa cells, thereby enhancing their proliferation rate [64]. Thus, it will be interesting to determine whether SRSF1 contributes to fine-tuning the activation of these pathways in PCa cells through the regulation of *MNK2* AS. 

Other splicing factors may also contribute to the altered splicing program of PCa cells. Indeed, the activity of several of these RBPs is modulated by signal transduction pathways that are frequently turned on in cancer, such as the PI3K/AKT and the RAS/ERK pathways (see also [66]). For instance, it was shown that activation of AKT downstream of the epidermal growth factor (EGF) receptor modulated the activity of the SR protein-specific kinases and phosphorylation of SR proteins, thereby affecting a large spectrum of AS events [67]. Similarly, the RAS/ERK pathway modulates a number of splicing factors involved in cancer, such as SAM68 [68] and the alternative splicing factor 45 (SPF45) [69], which in turn affect expression of splice variants that regulate cell motility, proliferation, and survival. Thus, it is likely that the examples reported above represent only a small picture of the overall contribution of AS and splicing factors to the wide heterogeneity in gene expression observed in PCa cells and patients.

## 10. Conclusions and Perspectives

AS is widely recognized as a powerful tool that eukaryotic cells employ to expand the coding potential and the plasticity of their genomes. The flexibility in the recognition of exons and introns within the transcription unit of the majority of human genes offers the possibility to compose many mRNA variants from each gene. Subtle changes in the cellular environment, or in external cues conveyed from the surrounding environment, may result in global changes in the transcriptome, which in part rely on the regulation of AS. An interesting observation is that apparently homogenous cell populations actually display large differences in gene expression. This was recently exemplified by studies that applied global RNA sequencing techniques to the analysis of single cell transcriptomes. After treatment of bone-marrow-derived dendritic cells (BMDCs) with an inflammatory cue, it was found that hundreds of key immune genes were differentially expressed by single cells. The heterogeneity in the response was particularly remarkable with regard to the splicing patterns expressed by these cells [70], suggesting that fine-tuning of AS regulation strongly contributes to the heterogeneity of a cell population. This aspect might be particularly relevant in the context of PCa, which is a neoplastic disease characterized by extreme heterogeneity and unpredicted response of patients to the therapy [1, 2]. The improvements in cell isolation techniques coupled to the higher sensitivity of the next-generation sequencing techniques may soon allow a highly detailed description of the transcriptome of patients, which might result in more personalized treatments.

The studies illustrated previously suggest that the upregulation of selected splicing regulators in PCa, such as SAM68, SRSF1, or DDX5, directly contributes to the phenotype by altering the splicing profile of key genes. Thus, these RBPs might represent potential therapeutic targets for intervention. Although blocking the activity of a given splicing factor is not necessarily an easy task, some examples in this direction have been provided. For instance, SAM68 can bind to RNA only as a dimer. By exploiting this requirement, it was shown that an RNA binding-defective SAM68 mutant exerted dominant negative effects on SAM68-mediated SMN2 splicing by associating with the endogenous protein and preventing its binding to the pre-mRNA [71]. This experiment suggests that small molecules interfering with SAM68 function might display therapeutic potential. As homodimerization is a prerequisite for RNA binding, one possibility is to target the SAM68 dimerization domain, which was restricted to a small region within its Gld1-Sam68-Grp33 (GSG) homology domain [72]. The potential value of targeting specific components of the splicing machinery in cancer cells is also suggested by the antioncogenic properties of natural compounds, such as spliceostatin A (SSA), in a variety of cancer cell models. SSA targets the splicing factor 3B subunit 1 (SF3B1) of the spliceosome, thus affecting a large number of splicing events concomitantly [73]. Perhaps, more specific drugs targeting splicing factors involved in subsets of oncogenic splicing events in cancer cells, as those described above, might represent more specific therapeutic approaches in the next future.

Although the extreme flexibility of AS regulation is prone to errors that may concur to neoplastic transformation [7, 8], it can also be exploited therapeutically. Indeed, examples of AS modulation in selected genes by administering splicing-correcting ASOs to cells have been reported. In some cases, this approach has also been challenged with a therapeutic application. One of the most remarkable examples is represented by the recovery of the phenotype observed in mouse models of Spinal Muscular Atrophy (SMA). This neurodegenerative disease is caused by inactivation of the *SMN1* gene and skipping of exon 7 in the highly homologous *SMN2* gene [74]. It was recently demonstrated that systemic injection of a chemically modified ASO restored *SMN2* splicing *in vitro* and *in vivo* and profoundly ameliorated the viability and phenotypic features of mice affected by a severe form of SMA [75]. Although cancer is caused by multiple alterations, thus limiting the application of gene-specific ASOs, it is conceivable that these tools could be used in combination with standard therapies to improve the clinical response of patients. For instance, an ASO that switched *BCL-X* splicing toward the proapoptotic variant was effective in sensitizing cancer cells to drug-induced apoptosis and to reduce growth of tumors in nude mice [30, 31]. A similar effect was obtained by switching expression of the *α* to the *β* variant of the signal transducer and activator of transcription 3 (*STAT3*) gene, which modulates multiple oncogenic pathways [76]. In this case, administration of a modified ASO targeted to a splicing enhancer induced expression of the endogenous STAT3*β* and an anti-oncogenic response *in vitro* and *in vivo* [76]. These studies suggest that modulation of AS with synthetic drugs is possible and has entered a therapeutic perspective. ASOs are particularly appealing in terms of high specificity and reduced side effects, as they may exploit their ability to anneal with specific sequences in the genome without affecting other features or target genes of the splicing factors involved in the oncogenic AS event. Thus, it is likely that these methods could be applied soon to the development of novel therapies aimed at fighting human cancers in which expression of specific oncogenic splice variants has been firmly confirmed.
